# ACTB and GAPDH appear at multiple SDS-PAGE positions, thus not suitable as reference genes for determining protein loading in techniques like Western blotting

**DOI:** 10.1515/biol-2021-0130

**Published:** 2021-12-13

**Authors:** Keyin Zhang, Ju Zhang, Nan Ding, Lucas Zellmer, Yan Zhao, Siqi Liu, Dezhong Joshua Liao

**Affiliations:** Department of Pathology, School of Clinical Medicine, Guizhou Medical University, Guiyang 550004, Guizhou Province, People’s Republic of China; Beijing Key Laboratory of Emerging Infectious Diseases, Institute of Infectious Diseases, Beijing Ditan Hospital, Capital Medical University, Beijing 100015, People’s Republic of China; Department of Medicine, Hennepin County Medical Center, 730 South 8th St., Minneapolis, MN 55415, United States of America; Key Lab of Endemic and Ethnic Diseases of the Ministry of Education of China in Guizhou Medical University, Guiyang 550004, Guizhou Province, People’s Republic of China; Beijing Genomic Institute, Building 11 of Beishan Industrial Zone, Tantian District, Shengzhen 518083, Guangdong Province, People’s Republic of China; Department of Clinical Biochemistry, Guizhou Medical University Hospital, Guiyang 550004, Guizhou Province, People’s Republic of China

**Keywords:** reference gene, beta-actin, GAPDH, SDS-PAGE, Western blotting, proteomics

## Abstract

We performed polyacrylamide gel electrophoresis of human proteins with sodium dodecyl sulfate, isolated proteins at multiple positions, and then used liquid chromatography and tandem mass spectrometry (LC-MS/MS) to determine the protein identities. Although beta-actin (ACTB) and glyceraldehyde-3-phosphate dehydrogenase (GAPDH) are 41.7 and 36 kDa proteins, respectively, LC-MS/MS identified their peptides at all the positions studied. The National Center for Biotechnology Information (USA) database lists only one ACTB mRNA but five GAPDH mRNAs and one noncoding RNA. The five GAPDH mRNAs encode three protein isoforms, while our bioinformatics analysis identified a 17.6 kDa isoform encoded by the noncoding RNA. All LC-MS/MS-identified GAPDH peptides at all positions studied are unique, but some of the identified ACTB peptides are shared by ACTC1, ACTBL2, POTEF, POTEE, POTEI, and POTEJ. ACTC1 and ACTBL2 belong to the ACT family with significant similarities to ACTB in protein sequence, whereas the four POTEs are ACTB-containing chimeric genes with the C-terminus of their proteins highly similar to the ACTB. These data lead us to conclude that GAPDH and ACTB are poor reference genes for determining the protein loading in such techniques as Western blotting, a leading role these two genes have been playing for decades in biomedical research.

## 1 Introduction

In 2012, we reported a bioinformatics study showing that the *ACTB* and *GAPDH* genes in the human and mouse genomes have a large number of intronless pseudogenes located on different chromosomes. The sequences of these pseudogenes are highly similar to the mRNA sequence of ACTB or GAPDH [1]. Because it is a general belief that the entire genomes are transcribed to RNA [2], these pseudogenes are likely transcribed, and their transcription is likely swayed by different developmental, physiological, or pathological conditions. Considering that the transcripts of these pseudogenes might be mistakenly detected along with the authentic ACTB or GAPDH RNA using reverse transcription polymerase chain reactions (RT-PCR), we suggested that biomedical researchers take extra caution when using these two genes as references in RT-PCR [1]. Besides this pseudogene issue, many studies have shown that the expression of ACTB and GAPDH varies among different developmental, physiological, and pathological situations, with several references adduced herein [3,4,5]. In congruence with its expression variation, GAPDH is known to have versatile functions, including membrane fusion, apoptosis, regulation of stability and transcription of RNA, and instability and repair of DNA, besides its canonical role in energy production [5,6,7]. The National Center for Biotechnology Information (NCBI, USA) database lists five mRNA variants and one long noncoding RNA of the human *GAPDH* gene; therefore, different functions of GAPDH may be elicited by different RNA variants or protein isoforms. ACTB has been reported to form fusion genes in some human neoplasms [8,9,10,11,12], and fusion genes involving *GAPDH* have also been reported in evolutionarily low organisms [13,14,15,16,17]. Actually, because of these weaknesses, searching for appropriate reference genes other than ACTB and GAPDH for PCR, RT-PCR, and Western blotting (WB) has become a prominent research area in the past decades. It is likely that different research purposes require different reference genes.

It is well known that most genes in the mouse, rat, and human genomes are expressed to multiple protein isoforms to meet various developmental, physiological, or pathological needs [2,18]. The mechanisms for protein multiplicity are themselves multiple, including alternative transcriptional initiation or termination to produce different RNA transcripts with longer or shorter 5′- or 3′-end, alternative splicing of a transcript to produce different mRNA variants with more or fewer exons, alternative uses of open reading frames (ORF) of an mRNA to produce different unrelated proteins, and alternative uses of in-frame start or stop codons within the same ORF to produce different protein isoforms with a longer or shorter N- or C-terminus [2]. In addition, single-nucleotide polymorphisms among different individual organisms and different genetic alterations (such as mutations) occurring at different pathological situations may affect transcription, splicing, or translation as well.

Currently, there lacks a simple but high-throughput technical approach to determine protein isoforms. High-throughput determination of protein expression is often achieved using a bottom–up approach of liquid chromatography and tandem mass spectrometry (LC-MS/MS), in which proteins are first enzymatically digested to short peptides before a LC-MS/MS procedure. The resulting MS data of each short peptide are then matched to a database of protein reference, which results in the amino acid (AA) sequence of the peptide, and in turn, the identity of the gene that produces the peptide-encompassing protein. Because this procedure uses a short peptide to predict the existence of a protein, it is referred to as “bottom–up.” Several years ago, we developed a simple tack to study protein multiplicity, in which proteins were first stratified based on their molecular weights using polyacrylamide gel electrophoresis (PAGE) in the presence of sodium dodecyl sulfate (SDS), followed by isolation of the proteins from the gel at a given position of the SDS-PAGE. These proteins with known molecular weights in the SDS-PAGE gel were then subjected to a routine LC-MS/MS procedure for their identification [19]. With this approach, we detected, unexpectedly, peptides of ACTB and GAPDH roughly at the 72, 55, 48, 40, and 26 kDa positions of SDS-PAGE [19,20,21], although ACTB and GAPDH proteins should be about 41.7 and 36 kDa, respectively. We herein report these ACTB and GAPDH data, along with some relevant bioinformatics analyses, and discuss the meaning behind these results.

## 2 Materials and methods

### 2.1 Protein sample preparation and SDS-PAGE

The proteomics part of this study included analyses of the ACTB- and GAPDH-related LC-MS/MS data derived from two separate experiments reported previously [19,20,22]. One experiment initially aimed to determine the identity of putative CDK4 isoforms at about 26 and 40 kDa positions of SDS-PAGE [19,22]. In this experiment, human breast cancer cell line MDA-MB231 and human embryonic kidney cell line HEK293 were routinely cultured at 37°C in an incubator with 5% CO_2_ in 10 cm dishes with a Dulbecco’s modified eagle medium containing 10% bovine fetal serum. The other experiment initially aimed to obtain global data about protein isoforms of human genes, in which human breast cancer cell lines MDA-MB231 and MCF7 were cultured in the same way as described above. In both experiments, cells at about 80% confluence were washed with 1× phosphate-buffered saline and then scraped in a lysis buffer [23] that contained 1× Protease Inhibitor Cocktail (Sigma-Aldrich, Inc, St. Louis, MS, USA), as described before [19,24]. After the cell lysate was centrifuged at 12,000 rpm for 15 min at 4°C, the supernatant was collected as the protein sample and determined for protein concentration with a bicinchoninic acid kit (Pierce, Rockford, IL, USA). The protein samples were diluted with a gel-loading buffer routinely used for WB, containing a final concentration of 2% SDS and 2% 2-mercaptoethanol. The proteins were boiled for 4 min, rapidly cooled on ice, and then loaded into a 10% SDS-containing polyacrylamide gel. To better separate and better detect the proteins, the gel was made using 10 × 10.5 cm glass plates included in the Hoefer SE260 vertical slab gel system (Hoefer Inc; http://www.hoeferinc.com/), which produced a gel that was 2 cm longer in the vertical direction than all gels made using the regular mini-gel casting systems of Hoefer and other companies.

**Figure 1:**
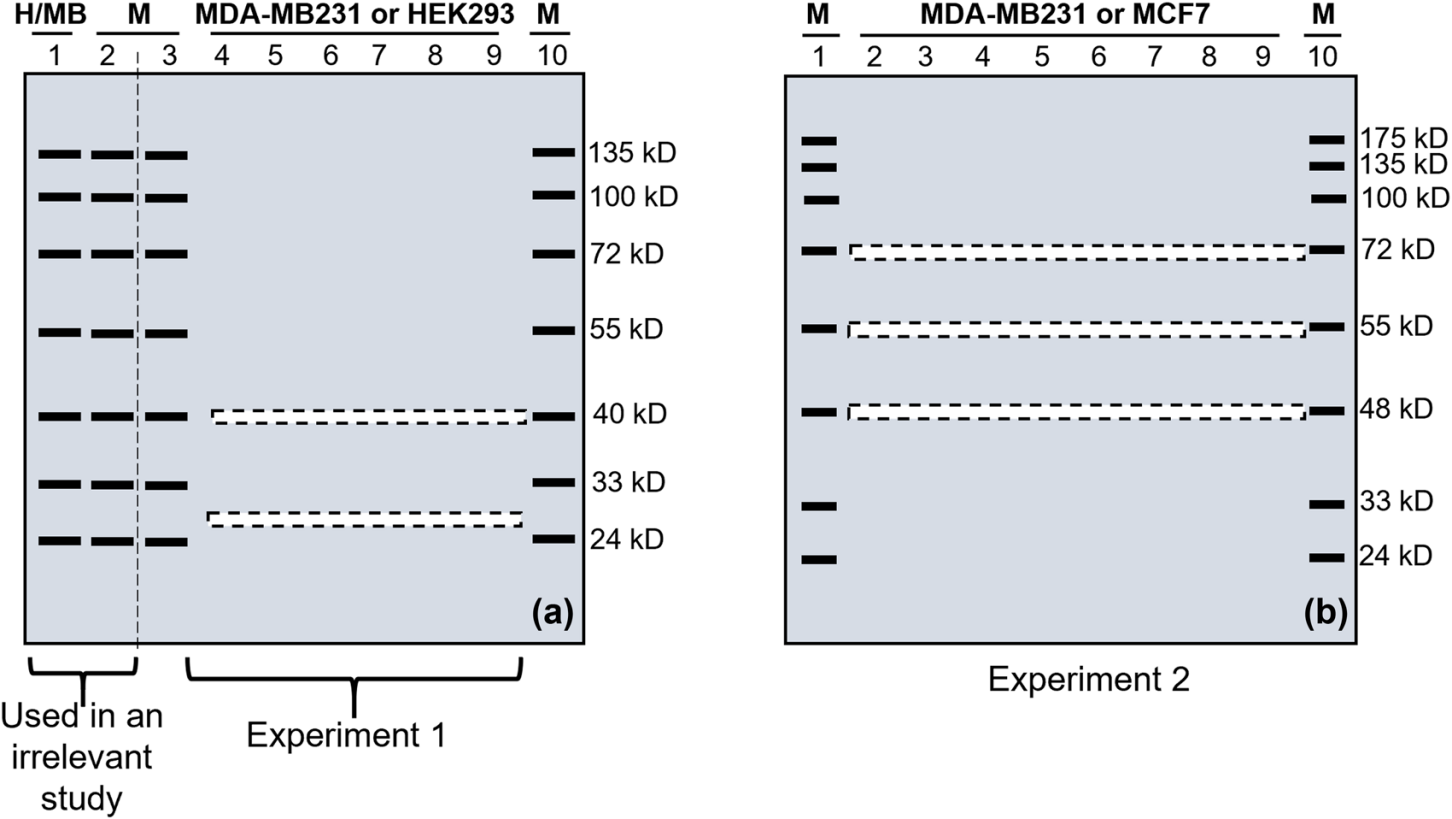
Illustration of excision of narrow stripes of gel after SDS-PAGE in two (a and b) experiments. (a) Two gels were made for the first experiment. The first well was loaded with proteins from HEK293 cells (H) in one gel and proteins from MDA-MB231 cells (MB) in the other gel. The second, third, and tenth wells of both gels were loaded with a prestained protein marker (M). The fourth to ninth wells of one gel were loaded with proteins from HEK293 cells, but these wells of the other gel were loaded with proteins from MDA-MB231 cells. After electrophoresis, both gels were cut into two parts along the vertical dashed line between the second and third lanes. The left part of both gels containing lanes 1 and 2 was used in WB to detect the CDK4 protein isoforms at the 40 and 26 kD positions, which was the initial purpose of this experiment but is irrelevant to the present study. The right part of both gels was used for this study, of which two narrow stripes (illustrated as dashed boxes) were excised at the 26 and 40 kD positions. (b) Two gels were also made in the second experiment. Although the first and the last wells of both gels were loaded with a prestained protein marker (M), the remaining wells were loaded with proteins from MDA-MB231 cells in one gel and proteins from MCF7 cells in the other gel. After electrophoresis, three narrow stripes shown as dashed boxes were excised from each gel at the 72, 55, and 48 kDa positions. All ten stripes from all the four gels of these two experiments were later used for LC-MS/MS analyses.

In the first experiment, the first well of one gel was loaded with 100 µg of the proteins from HEK293 cells whereas the first well of the other gel was loaded with 70 µg of the proteins from MDA-MB-231 cells. The second, third, and tenth wells of both gels were loaded with a prestained protein marker that contained bands at the positions indicated in Figure 1a. The remaining fourth to ninth wells of one gel were loaded with 70 μg of the proteins from the MDA-MB231 cells, whereas these wells of the other gel were loaded with 100 µg of proteins from the HEK293 cells. The two gels were electrophoresed simultaneously using the same power supply, and electrophoresis was stopped when the lowest (11 kDa) marker just ran out of the gel. In the second experiment, the first and last wells of both gels were loaded with a prestained protein marker, whereas each remaining well was loaded with 50 µg of the proteins from MCF7 cells in one gel and 60 µg of proteins from MDA-MB231 cells in the other gel (Figure 1b). Electrophoresis of the proteins was performed as described above.

### 2.2 Excision of narrow stripes of gel

In the first experiment, the two gels were first cut vertically with a surgical blade along the dashed line between the second and third lanes, as illustrated in Figure 1a. The part containing the first two lanes was used for a separate WB analysis to detect the CDK4 protein isoforms at about 26 and 40 kDa positions [22], which was the initial purpose of this experiment but is irrelevant to the current study. Later, guided with two rulers along with the prestained marker at the third and tenth lanes, we excised a narrow stripe (about 2 mm in width) near the 26 kDa position of the fourth to ninth lanes of each gel, and then another narrow stripe at the 40 kDa position shown by the prestained marker (Figure 1a).

In the second experiment, a 2 mm stripe of gel was excised at the 72, 55, and 48 kDa positions shown by the corresponding prestained protein marker in the first and last lanes (Figure 1b). These positions were selected after carefully considering many technical issues: first, we had prestained protein markers showing these positions, which allowed us to excise a narrow gel stripe at the correct molecular weight. Second, this 48–72 kDa range resides in the middle of the 10% gel made using most mini-gel-casting systems. This middle range still leaves us with large regions below the 48 kDa and above the 72 kDa. Third, proteins with very large molecular weights, such as larger than 150 kDa, cannot be well separated in a 10% gel.

### 2.3 LC-MS/MS

As described before in detail refs. [19,20], the protein-containing gel stripes were dehydrated with escalating concentrations of acetonitrile (ACN). The in-gel proteins were reduced and alkylated with 10 mM dithiothreitol and 55 mM iodoacetamide, followed by digestion with trypsin at 37°C for 16 h [22]. The tryptic peptides were then extracted from the gel with ACN containing 0.1% formic acid (FA), vacuum-dried, and dissolved in 0.1% FA. The peptides were delivered onto a nano-reverse phase column (5 μm Hypersil C18, 75 mm × 100 mm; Thermo Fisher Scientific, Waltham, MA, USA) and eluted with escalating (50–80%) concentrations of ACN for 60 min at a speed of 400 nL/min. Different fractions of the eluate were injected into a Q-Exactive mass spectrometer (Thermo Fisher Scientific, Waltham, MA, USA) preset in a positive ion mode and in a data-dependent manner with a full MS scan ranging from 350 to 2,000 m/z. High-energy collisional dissociation was used as the MS/MS acquisition method. Raw MS/MS data were converted into a mascot generic format (MGF) using Proteome Discoverer 1.2 (Thermo Fisher Scientific, Waltham, MA, USA). The exported MGF files were searched with Mascot v2.3.01 in a local server against the human SwissProt database. All searches were performed with a tryptic specificity allowing for a one-time missed cleavage. Carbamidomethylation was considered as a fixed modification, whereas oxidation (M) and Gln- > pyro-Glu (N-term Q) were considered as variable modifications. The mass tolerance for MS and MS/MS was 15 ppm and 20 mmu, respectively. Proteins with false discovery rates <0.01 were further analyzed.

### 2.4 Retrieval and analyses of bioinformatics information

The RNA and protein sequences were retrieved from the NCBI, USA website (https://www.ncbi.nlm.nih.gov/gene/). ORF of an RNA and molecular weight of the ORF-encoded protein were determined using the DNAstar software (https://www.dnastar.com/). Sequence alignment was performed using the Blast function of NCBI. Distance tree analysis of RNA sequences was also performed using the Blast function, with the figure redrawn to make the tree clearer.

### 2.5 Calculation of the total coverage rate and the unique coverage rate

The LC-MS/MS procedure generated two basic sets of datasheets, annotated as “proteingroups” and “psms,” respectively. The “proteingroups” datasheet contains “coverage” data (column D in the Table S1), which is the ratio of the total number of AAs in all LC-MS/MS-identified peptides to the total number of AAs in the annotated protein of a particular gene. This coverage is coined herein as “the total coverage rate.” The sequence of each identified peptide is given in the “psms” datasheets (Tables S2 and S3). For many genes, including *ACTB*, some LC-MS/MS-identified peptides are not unique to the annotated protein of the particular gene but, instead, are also shared by protein(s) of one or more other genes, which are referred to as “common peptides.” We retrieved the sequence of each identified peptide, common or unique, from the “psms” datasheet for GAPDH or ACTB, and mapped the sequence onto the full-length protein of GAPDH or ACTB. We then calculated the total coverage rate, which is the ratio of the total AAs of both common and unique peptides to the total AAs of the full-length GAPDH or ACTB protein. We also calculated the “unique coverage rate,” which is the ratio of the total AAs of the unique peptides to the total AAs in the full-length GAPDH or ACTB protein. A higher unique coverage rate indicates a higher possibility of the presence of the protein in the studied position of the SDS-PAGE gel.

## 3 Results

### 3.1 The number of genes proteins of which are detected

The “proteingroup” datasheet for each gel stripe (Table S1) lists each gene’s name and the accession number of the protein identified. From the datasheets, we calculated the total number of genes identified (Table 1).

**Table 1:** Numbers of genes proteins of which are detected

MDA-MB231		MCF7		HEK293	
Stripe (kDa)	Number	Stripe (kDa)	Number	Stripe (kDa)	Number
72	679	72	490	40	968
55	750	55	390	26	1,096
48	765	48	470		
40	376				

### 3.2 RNAs and proteins of GAPDH listed in the NCBI

**Figure 2:**
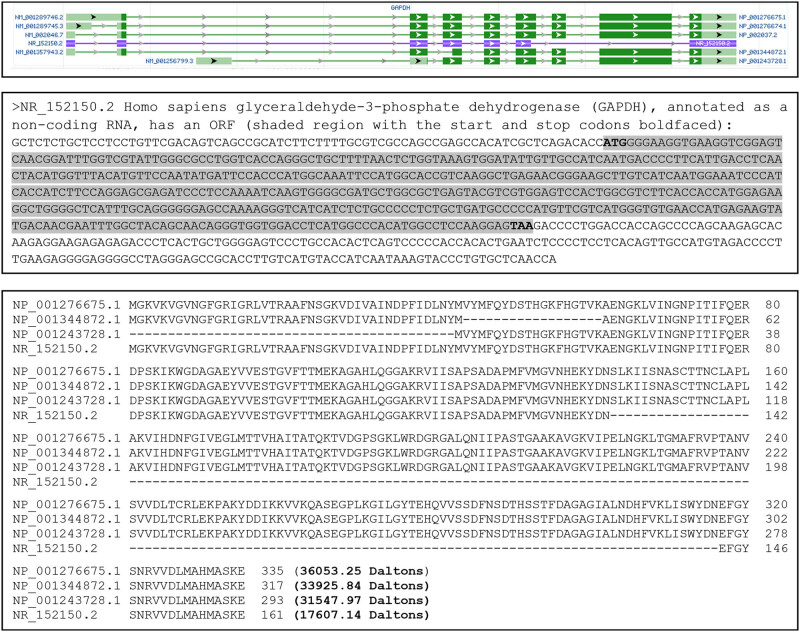
RNA variants and protein isoforms of the human *GAPDH* gene. An image copied from the NCBI database shows six RNA variants of *GAPDH* (top panel). The NR_152150.2 is annotated as a noncoding RNA, but, according to our analysis, it encodes an ORF for a GAPDH isoform of 161 AAs, shown as the shaded sequence with its ATG start codon and TAA stop codon boldfaced (middle panel). Because three of the five mRNA variants, that is, NM_0012589746.2, the NM_001289745.3, and the NM_002046.7, encode the same protein, the six RNAs encode a total of four protein isoforms, with their similarities and disparities as well as their numbers of AAs and molecular weights shown in the bottom panel.

The NCBI database lists six RNA variants of the human *GAPDH* gene, including five normalized mRNA variants annotated as NM_ sequences (NM_001289746.2, NM_001289745.3, NM_0020467, NM_001357943.3, and NM-00125799.3) and one predicted noncoding RNA annotated as a NR_ sequence (NR_15150.2). Five of the six, including the noncoding variant, are derived from alternative splicing, whereas the remaining one is derived from alternative initiation of transcription from the first intron of the NM_001289746.2 sequence (Figure 2, top panel). Three proteins (NP_001276675.1, NP_001276674.1, and NP_002037.2 encoded, respectively, by NM_001289746.2, NM_001289745.3, and NM_002046.7) have the same AA sequence, with the NP_00127665.1 protein shown as a representative in the middle panel of Figure 2 and considered herein as the full-length one. Compared with this full-length sequence, protein NP_1344872.1 lacks 18 AAs because its exon 4 is shorter (Figure 2, top panel), whereas protein NP_001234728.1 lacks the N-terminal 42 AAs because the alternative initiation of transcription leads to the use of a different start codon for translation (Figure 2, top and middle panels). Although NR_152150.2 is annotated by the NCBI as a noncoding RNA, our bioinformatics analysis identified an ORF encoding a GAPDH protein isoform of 161 AAs, which is constituted by the N-terminal 142 AAs and the C-terminal 19 AAs of the full-length protein. Therefore, the human *GAPDH* gene has at least four protein isoforms based on the NCBI information, with their similarities and disparities as well as their theoretical molecular masses shown in the bottom panel of Figure 2.

### 3.3 Identification of ACTB-homologous genes

**Figure 3:**
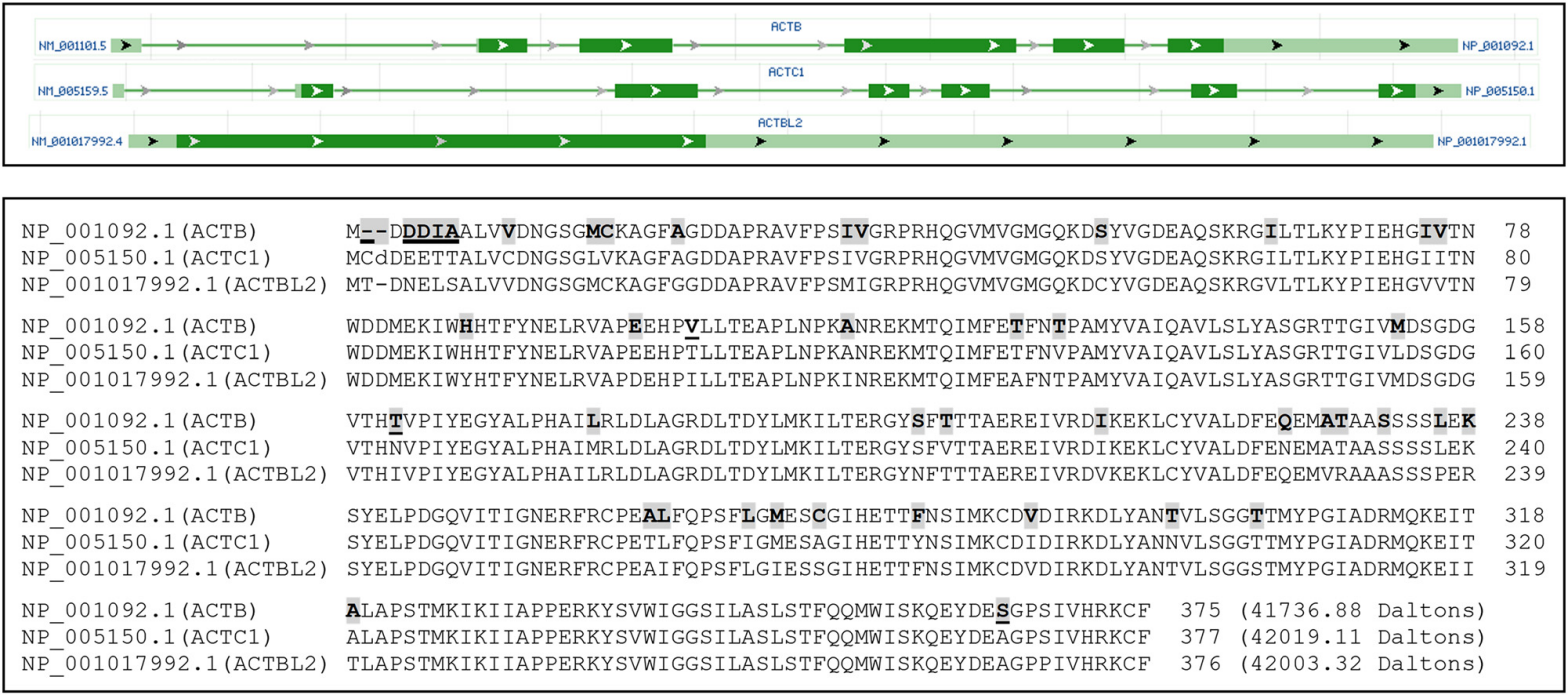
Similarities of ACTB to ACTC1 and ACTBL2. An image copied from the NCBI database shows that ACTB, ACTC1, and ACTBL2 have only one RNA, with the ACTBL2 being a one-exon gene (top panel). Alignment of ACTB, ACTC1, and ACTBL2 proteins shows that their AA sequences are highly similar (bottom panel). The AAs in ACTB that differ from either ACTC1 or ACTBL2 are shaded, whereas the AAs in ACTB that differ from both ACTC1 and ACTBL2 are shaded and underlined.

**Figure 4:**
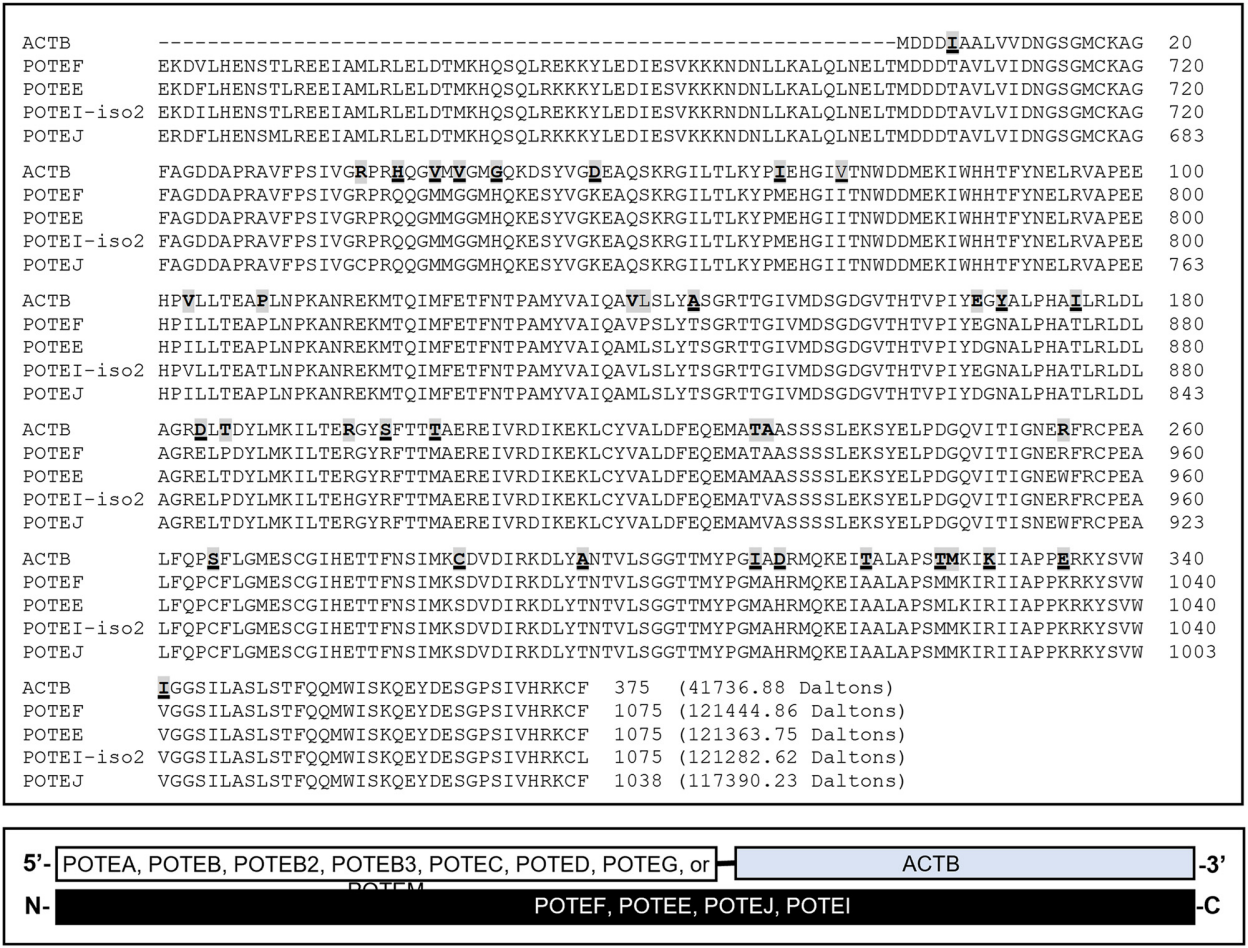
Similarities of ACTB to the C-terminal part of several POTE proteins. Alignment of the ACTB protein with the POTEF, POTEE, and POTEJ proteins as well as the protein isoform 2 of POTEI shows that ACTB is highly similar to the C-terminal part of these four POTE proteins (top panel). The AAs in ACTB that differ from only one, two, or three of the four POTE proteins are shaded, whereas the AAs in ACTB that differ from all of the four POTE proteins are shaded and underlined. It seems that these four *POTE* genes might be formed as fusion genes between the 3′-end of the *POTEA*, *POTEB*, *POTEB2*, *POTEB3*, *POTEC*, *POTED*, *POTEG*, or *POTEM* gene (which do not have an ACTB-element) and the 5′-end of the *ACTB* gene (bottom panel).

**Figure 5:**
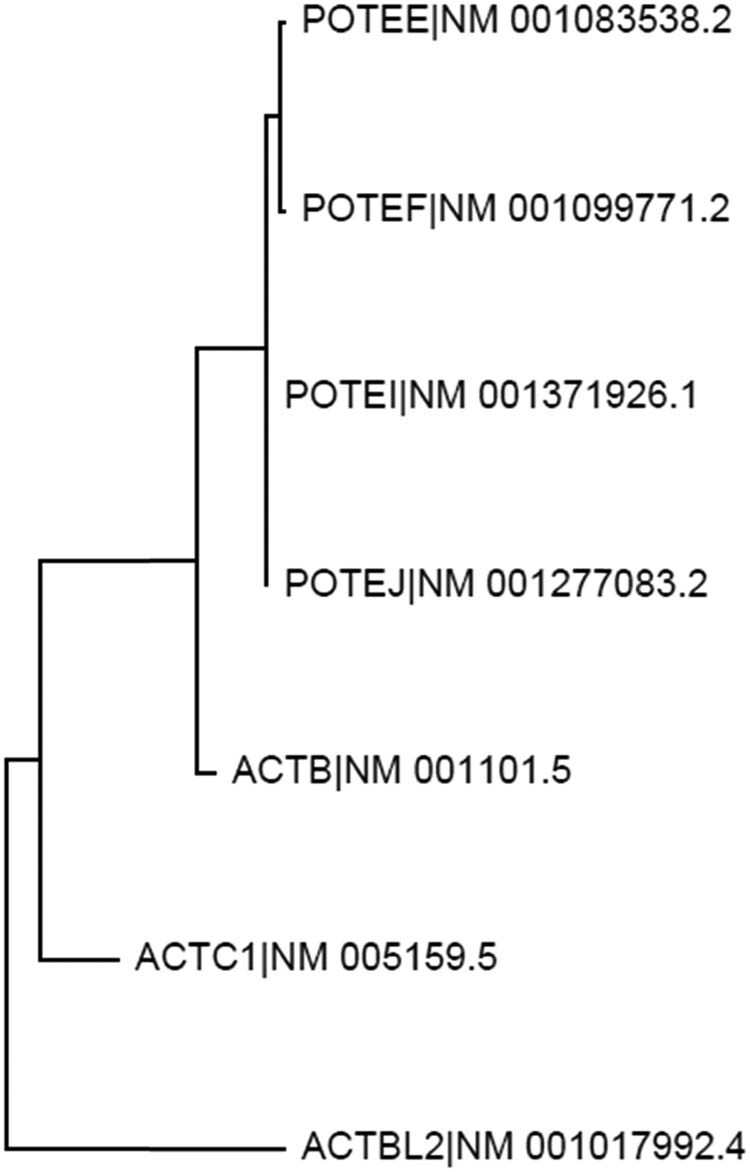
Evolutionary relationship among the *ACTB*, *ACTC1*, *ACTBL2*, and several *POTE* genes. Distance tree, resulting from analysis of the evolutionary distances among the mRNAs of ACTB (NM_001101.5), ACTC1 (NM_005159.5), ACTBL2 (NM_001017992.4), POTEF (NM_001099771.2), POTEE (NM_001083538.2), POTEI (NM_001371926.1) and POTEJ (NM_001277083.2), suggests that ACTB may be evolutionarily closer to the four *POTE* genes than to *ACTC1* and then to the *ACTBL2*.

The NCBI database lists only one ACTB RNA, which is an mRNA encoding a 375-AA protein (NP_001092.1). Our “psms” data (Tables S2 and S3) show that several identified peptides of ACTB are shared by ACTC1 and ACTBL2, which are two other ACT family members. The alignment of protein sequences of ACTB, ACTC1, and ACTBL2 confirms this finding (Figure 3). Moreover, some identified peptides of ACTB are shared by the C-terminus of proteins from several *POTE* genes, namely *POTEF*, *POTEE*, *POTEI*, and *POTEJ* (Figure 4, top panel). The *POTE* gene family still has seven other members that encode longer mRNAs, including *POTEA*, *POTEB*, *POTEB2*, *POTEC*, *POTED*, *POTEG*, and *POTEM*, besides a pseudogene (*POTEKP*) that codes for a noncoding RNA. The proteins from the seven *POTE* genes share only the N-terminal region with POTEF, POTEE, POTEJ, and POTEI, and thus do not have any similarity to the ACTB protein. We surmise that during evolution, the 3′-end of one of the several shorter *POTE* genes fused to the 5′-end of the ACTB, and later this fusion gene evolved to the other three ACTB-containing *POTE* genes (Figure 4, bottom panel). Interestingly, analysis of the evolutionary distances between the mRNAs of *ACTB*, *ACTC1*, *ACTBL2*, and the four *POTE* genes reveals that ACTB is evolutionarily closer to the four *POTE*s than to *ACTC1* and *ACTBL2* (Figure 5). Therefore, ACTB is likely to evolve to ACTC1 and then to ACTBL2 first, and later to POTEF, POTEE, POTEJ, or POTEI. In line with this inference, the ACTB protein has a total of 39 different AAs compared to ACTC1 or ACTBL2 (Figure 3) but only has 36 different AAs compared to the ACTB-containing region of the four TOPE proteins (Figure 4).

### 3.4 Detection of GAPDH and ACTB at the 72-, 55-, 48-, 40-, and 26 kDa positions of SDS-PAGE

**Figure 6:**
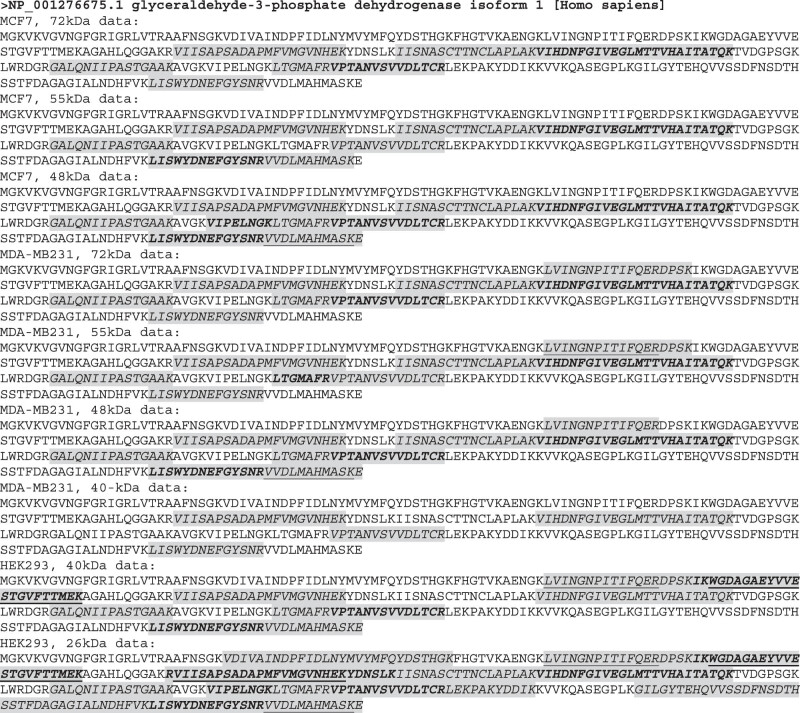
Location of LC-MS/MS-identified peptides on the Wt GAPDH protein. LC-MS/MS-identified peptides (shaded and italicized regions) are mapped onto the full-length GAPDH protein. Some long-identified sequences are actually formed by several consecutive identified peptides with boldfaced sequence(s) to segregate one from another. Sometimes a peptide was identified as a slightly longer or shorter version of another one; in this case, the shorter version is underlined. For instance, both “VVDLMAHMASKE” and “VVDLMAHMASK” are identified, with the underlined one lacking the “E.”

Although the full-length GAPDH is about 36 kDa (Figure 2), our LC-MS/MS analyses identified short peptides of GAPDH from both MDA-MB231 and MCF7 cells at the 72, 55, and 48 kDa positions, from both MDA-MB231 and HEK293 cells at the 40 kDa position, and from HEK293 cells at the 26 kDa position. All of the identified peptides are unique to GAPDH. We mapped these peptides onto the full-length GAPDH protein and found that each of the four GAPDH isoforms contained at least two unique peptides (Figure 6). We calculated the coverage rate at each gel position for each cell line and found that all of the rates matched with the rates provided in the “proteingroups” datasheet (Table 2 and Table S1). Interestingly, the HEK293 cells at the lowest position, that is, at 26 kDa, show the highest coverage rate, reaching 76.72% (Table 2 and Figure 6). It is worth mentioning that, because the LC-MS/MS approach used short peptide(s) to predict the existence of a protein, the peptides detected in the same gel stripe may not necessarily belong to the same isoform, as it cannot be excluded that they belong to different known or unknown isoforms that have similar molecular weights and thus appear roughly at the same position.

**Table 2:** Coverage rates of identified peptides

Cell	Position (kDa)	GADPH	ACTB				ACTC1	ACTBL2
		(All unique) (%)	Total (%)	Unique (%)	Common (%)	U/T (%)	Unique (%)	
MCF7	72	33.43	48.80	21.33	21.47	43.72	4.24	12.76
	55	34.63	48.00		26.67	44.44		Undetected
	48	39.40	46.13		24.80	46.24		12.76
MDA-MB231	72	38.81	56.53	28.80	27.73	50.94	4.24	7.97
	55	38.81	65.33^a^	33.07	32.26	50.61	4.24	7.97
	48	41.19	65.87	33.07	32.80	50.20	9.81	18.35
	40	21.79	64.80^b^	33.07	31.20	51.45	4.24	7.97
HEK293	40	44.18	65.33	26.40	38.93	40.41	15.38	10.63
	26	76.72	45.33	25.06	20.27	55.28	4.77	Undetected

Note: As ACTC1 and ACTBL2 are not the focuses of this study, only their unique coverage rates are calculated. ^a^The data in the “proteingroups” datasheet is 61.33%, slightly lower than the rate we calculated. ^b^The data in the “proteingroups” datasheet is 70.00%, higher than what we calculated. U/T, ratio of the unique coverage rate to the total coverage rate.

**Figure 7:**
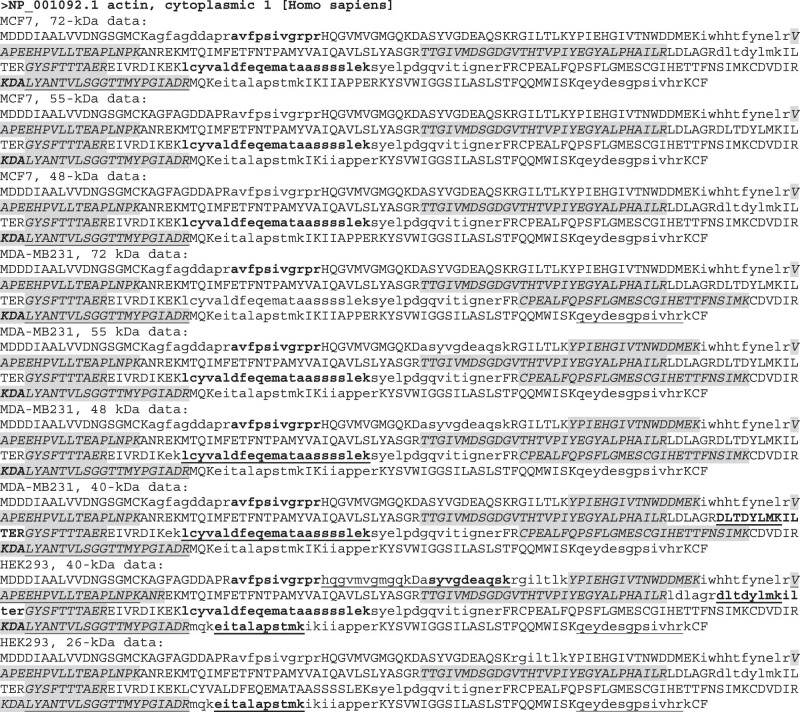
Location of LC-MS/MS-identified peptides on the ACTB protein. LC-MS/MS-identified peptides are mapped onto the ACTB protein, with the shaded and italicized regions being the unique peptides and the lowercase regions being the common peptides. Some long-identified sequences are actually formed by several consecutive identified peptides with boldfaced sequence(s) to segregate one from another. Sometimes a peptide was identified as a slightly longer or shorter version of another one; in this case, the shorter version is underlined. For instance, both “QEYDESGPSIVHRK” and “QEYDESGPSIVHR” are identified, with the underlined one lacking the “K.”

Identified peptides of ACTB included both unique and nonunique ones. The nonunique ones, referred to as “common” herein, are shared by the ACTC1, ACTBL2, POTEF, POTEE, or POTEJ protein, or with protein isoform 2 of the POTEI. These peptides are the clues leading us to discover the similarity of ACTB to *ACTC1*, *ACTBL2*, and the four ACTB-containing *POTE* genes. We mapped all identified peptides onto the full-length ACTB protein and calculated not only the coverage rates by the unique peptides but also the total coverage rate by both common and unique peptides (Figure 7). Most of the total coverage rates matched the rates given in the “proteingroups” datasheet (Table 2 and Table S1), but for unknown reasons, two of our calculations differ slightly (65.33 vs 61.33% and 64.80 vs 70.00%; Table 2). Nevertheless, all of the total coverage rates are high for different cell lines at different SDS-PAGE positions. The unique coverage rates are also high, varying between 21.33 and 33.07%, and contribute to more than 40% of the total coverage rates (Table 2).

Some peptides, and some AAs in a peptide, are identified in some cell lines at some SDS-PAGE positions but not in or at some others. We counted those AAs that have been identified in at least one cell line at one position to obtain the theoretical maximal-identified AAs, which is 252 AAs for ACTB. Because the ACTB protein has 375 AAs, its theoretical maximal-total-coverage rate should be 252/375, that is, 67.20%. None of our calculations reaches this theoretical maximum, but many are close (Table 2). In a similar way, we obtained the theoretical maximal-unique-coverage rate for ACTB, which is 33.07% and has actually been obtained in the MDA-MB231 and HEK293 cells for most positions, but not in the MCF7 cells at any position (Table 2), likely due to some technical reasons.

### 3.5 Possible translational mechanisms for the generation of isoforms

**Figure 8:**
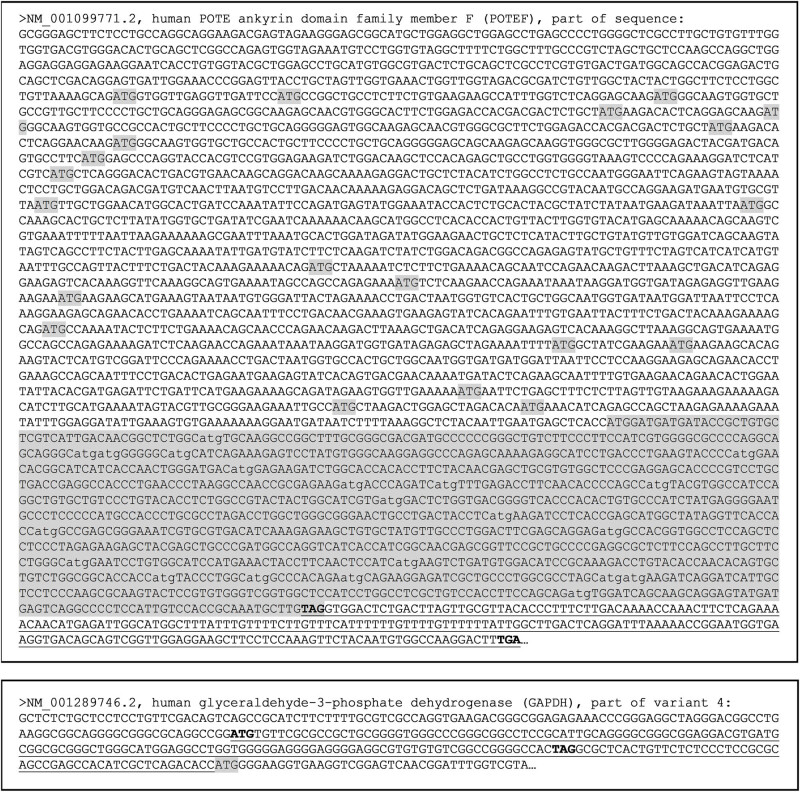
Depiction of some mechanisms for N- or C-terminal extension or for N-terminal truncation of a protein, with POTEF and GAPDH as examples. Top-panel: Part of the *POTEF* mRNA sequence, with all in-frame ATG start codons, and the *ACTB*-homologous region shaded. If translation starts with any one of the downstream ATGs, an N-terminally truncated POTEF isoform will be generated that may be mistakenly detected as a larger ACTB with certain techniques such as WB. However, if a mutation occurs in the annotated TAG stop codon (boldfaced), translation will be extended to a downstream TGA stop codon (boldfaced), producing a POTEF isoform with additional 73 AAs encoded by the underlined sequence, which may also be mistakenly detected as an ACTB isoform. Bottom-panel: Part of the 5′-sequence of a *GAPDH* mRNA showing an upstream ORF (with its ATG start codon and TAG stop codon boldfaced) that is in-frame with the ORF of *GAPDH*. If a mutation occurs in the TAG stop codon of the upstream ORF, translation of the upstream ORF will be extended to the ATG start codon (shaded) of the *GAPDH*, producing a GAPDH isoform with additional 64 AAs at the N-terminus encoded by the underlined sequence.

As we have discussed previously [2,22,24,25,26], utilization of a downstream start codon in a mRNA for translation to generate a protein isoform with a shorter N-terminus is very common, with a generation of some smaller isoforms of c-Myc, P53, and RB as epitomes [27,28,29]. Theoretically, this mechanism may also be used in the translation of *GAPDH*, *ACTB*, *POTEE*, *POTEF*, *POTEI*, and *POTEF* to generate shorter isoforms since all of these genes have many in-frame ATGs, as exemplified by the *POTEF* shown in Figure 8 (top panel). Other in-frame start codons besides ATG also exist but are not analyzed herein to avoid overwhelming the figure. These start codons include CTG that is often used for protein translation, such as for the generation of a c-Myc or PTEN isoform [27,30]. Single-nucleotide polymorphisms and, in pathological situations, single-nucleotide mutations may alter the canonical start codon leading to translation initiated from a downstream start codon as well. In addition, if there is an upstream ORF, its translation may be extended to the annotated ORF, engendering a longer N-terminus (Figure 8, bottom panel). However, if such polymorphisms or mutations occur at the annotated stop codon, translation may be extended to a downstream one, resulting in an isoform with a C-terminal extension (Figure 8, top panel).

### 3.6 Some posttranslation modifications that may affect protein migration in SDS-PAGE

After translation, proteins are often subjected to various chemical modifications that can affect their migration in SDS-PAGE. We, therefore, calculated the changes in molecular mass that may be caused by some common chemical modifications (Table 3). For instance, one cholesterolation, glycosylation, glycosylphosphatidylinositol (GPI) anchor, ubiquitination, and SUMOylation can, theoretically, increase the molecular mass of 0.4, 0.45–3.3, 2–3, 8.6, and 12 kDa, respectively (Table 3). In contrast, some other types of chemical modification change the molecular mass only slightly. Some modifications, such as phosphorylation, alter not only the molecular mass but also the electronic charge of a protein and thus may accelerate or decelerate migration of the protein in SDS-PAGE, depending on whether the migration is swayed more significantly by the change in the molecular mass or in the electronic charge. Many types of chemical modifications, such as phosphorylation, can simultaneously occur to many AAs of a protein, collectively making a huge impact on its migration in SDS-PAGE. Moreover, polyubiquitination, poly-SUMOylation, polyglycylation, polyglutamylation, and polyamination can occur as a chain, most of which have been well studied for tubulin as an example [30,31,32]. Any of these chains can greatly slow down protein migration.

**Table 3:** Some posttranslational chemical modifications of proteins that affect protein migration in SDS-PAGE

Modification	Modified	Modification	Modified (kDa)
Ubiquitination	[8.56]_1-*n*_ (*n* ≥ 20)	Heme C	0.62
SUMOylation	[12]_1-*n*_^a^ (*n* ≥ 10)	Flavin (FMN/FAD)	0.46/0.79
FATylation	[18]_1-*n*_ (*n* ≥ 3)	Phosphopantetheine	0.34
NEDDylation	[6,7,8,9,10]_1-*n*_ (*n* ≥ 5)	Retinylidene	0.28
ISGylation	17^a,b^	Lipoylation	0.19
ADP-ribosylation	[0.54]_1-*n*_ (*n* ≥ 200)	GPI (GPI-like) anchor	2-3
Adenylylation	0.33	Cholesterolation	0.4
Glycosylation	[0.13-0.31]_3-*n*_ (*n* ≥ 30)	Geranylgeranylation	0.27
Glycation	0.16^c^	Palmitoylation	0.24
Polyglycylation	[0.06]_1-*n*_ (*n* ≥ 40)	Farnesylation	0.21
Polyglutamylation	[0.13]_1-*n*_ (*n* ≥ 6)	Myristoylation	0.19
S-Glutathionylation	0.31	Diphthamidation	0.30
Arginylation	0.16	ETA phosphoglycerylation	0.27
Iodination	0.13	Phosphoglycerylation	0.17
Succinylation	0.10	Phosphorylation	1-2

Note: Most of the modifications increase the molecular weight of the protein and thus decelerate migration of the protein in SDS-PAGE, but some, such as phosphorylation, may sometimes accelerate migration when they change electronic charge of the protein more significantly than the molecular weight. (a) Both of the modification moieties could form mixed chains with ubiquitin; (b) there have not been poly-ISG15 chains or enzymes reported so far that are involved in the formation of poly-ISG15 chains; and (c) the glycation products could be further modified to produce inter-protein cross-links, carboxymethyl lysines, and other intermediates. SUMO-, small ubiquitin-related modifier; FAT-, HLA-F adjacent transcript; NEDD-, neural precursor cell expressed, developmentally down-regulated; ISG-, interferon-stimulated gene; ADP, adenosine diphosphate; FMN, flavin mononucleotide; FAD, flavin adenine dinucleotide; GPI, glycosylphosphatidylinositol; and ETA, ethanolamine.

## 4 Discussion

In this study, we reported that peptides of *ACTB* and *GAPDH* could be detected, using an LC-MS/MS approach, in protein samples from several human cell lines at the 72-, 55-, 48-, 40-, and 26 kDa positions of SDS-PAGE, like proteins of a large number of other genes reported previously [19,20,21]. One simple explanation is that *ACTB*, *GAPDH*, and these other genes are expressed as multiple protein isoforms as it is known that most human genes can produce multiple protein isoforms [2,18]. However, since LC-MS/MS uses short peptide(s) to predict the existence of a whole protein, it is possible that some detected peptides may not be derived from the authentic genes but, instead, are derived from other genes that contain element(s) of the authentic gene. By analyzing the sequences of our LC-MS/MS-identified peptides, we inadvertently found that POTEE, POTEF, POTEI, and POTEJ proteins have a region highly similar to the ACTB, which strengthens this possibility. It is worth mentioning that these “other genes” may be currently unknown or unannotated, as the human genome encompasses a huge number of unknown or unannotated genes [33]. In addition, in pathological situations, fusion genes may be formed, such as the *ACTB-FOSB* and *ACTB-GLI1* fusion genes found in some neoplasms [8,9,10,11,12]. More intricately, one mRNA can be polycistronic, encoding two or more proteins that are unrelated, and the human genome may produce a colossal number of polycistronic mRNAs that encode unannotated proteins [34].

The GAPDH and ACTB proteins detected at the 40 kDa position may be the wild type (Wt) form of 36 and 41.7 kDa, respectively, as protein migration in an SDS-PAGE gel can be affected by various factors, and most prestained protein markers are not very accurate. GAPDH [35,36] and ACTB [37,38] are known to be subjected to many types of posttranslational modifications. Therefore, theoretically, a combination of multiple types of chemical modifications, such as the formation of polyubiquitin, poly-SUMO, polyglycylation, polyglutamylation, or polyamination chain, can shift the Wt GAPDH and ACTB to the 48 kD, 55 kDa, and even 72 kDa positions. Other possibilities for the detection of GAPDH and ACTB at a higher position include that they are unknown isoforms, or they belong to other genes with a GAPDH- or ACTB-element.

Although the detected GAPDH and ACTB peptides at the 26 kDa position may be degraded fragments, it remains possible that their detection indicates the existence of unknown isoforms that are smaller than the Wt protein, resulting from mechanisms such as translation initiated from a downstream start codon, as depicted in Figure 8. If this scenario also occurs to one of the four ACTB-element-containing TOPE proteins, a smaller ACTB-like TOPE protein may be produced with a molecular weight varying from several kDa to 120 kDa (the molecular weight of the Wt POTE).

Besides the above-described scenarios that may occur physiologically, mutations may occur in many pathological situations, including in immortalized cell lines, leading to the generation of larger or smaller protein isoforms of a gene via different mechanisms. For example, if a mutation occurs to the stop codon of the upstream ORF in the GAPDH mRNA shown in Figure 8, translation of this ORF may be extended to the annotated ORF, yielding an N-terminal-extended GAPDH isoform. Similarly, if a mutation occurs to the annotated stop codon, translation will be extended downstream, resulting in a C-terminal-extended isoform (Figure 8).

For both ACTB and GAPDH, some peptides were detected in some cell lines at some positions but not in or at some others (Figures 6 and 7). The reasons could be technical or biological. The absence of a peptide in a cell line at an SDS-PAGE position may be because the cell line does not express the isoform containing the peptide region. Therefore, identification of an absent region by mapping the detected peptides onto the Wt protein sequence, as shown in Figures 6 and 7 for ACTB and GAPDH, may provide us with clues for the identification of unknown protein isoforms that have a specific region deleted due to such as the omission of particular exon(s), for example.

Most human genes produce multiple protein isoforms [2,18], and therefore researchers should often see not only the expected band but also additional band(s) on a WB membrane. That is indeed the case in reality. However, when multiple bands appear, a common but hardly mentioned practice is to cut off the unexpected band(s) from the membrane and present only the expected one, with the assumption, sans any supporting evidence, that the unexpected band(s) are nonspecific. Antibody supplier companies are often blamed for selling “lousy, not specific enough” antibodies. To avoid being blamed, most suppliers try hard to select and supply those antibodies that recognize only the expected protein form, usually the Wt or the canonical one. This is technically feasible as different isoforms may manifest different conformations inside the antibody-producing animal, making B lymphocytes produce some antibodies that recognize only one isoform but not the others. Although this compromise between researchers and antibody suppliers may lead to biased, somewhat misleading conclusions, it, unfortunately, has made it more difficult to find commercial antibodies that can recognize multiple isoforms rather than to find those recognizing only the Wt protein in general [18,33]. Indeed, many, probably most, commercial GAPDH and ACTB antibodies recognize only the Wt protein, although there still are some published WB data of GAPDH [39,40,41,42] and ACTB [39,42,43,44,45] showing two or three bands on the membrane. Many published WB results that have only a single band detected may be due to this compromise, although there certainly are many cases in which the gene of interest does indeed produce only a single isoform (e.g., the Wt form) in the given cell type at the given situation. Although primary antibodies that recognize only a single isoform are useful, those that recognize multiple isoforms and thus seem less specific may provide us with a more global picture of the protein products of the gene in question.

In summary, our LC-MS/MS analyses identified multiple peptides of ACTB and GAPDH at multiple SDS-PAGE positions, which raises a few questions, such as whether these two genes express some unknown protein isoforms. GAPDH has four protein isoforms, including one encoded by an RNA variant annotated by the NCBI as a noncoding one, whereas ACTB is highly similar in AA sequence to ACTC1, ACTBL2, and proteins of four POTE family members. Moreover, it is known that GAPDH has versatile functions and that both ACTB and GAPDH may be subjected to many types of posttranslational modifications. These lines of information lead us to a somewhat provocative conclusion that ACTB and GAPDH are not suitable for serving as the reference genes for protein loading in such techniques as WB, a leading role these two genes have been playing for decades in biomedical research.

## References

[1] Sun Y, Li Y, Luo D, Liao DJ. Pseudogenes as weaknesses of ACTB (Actb) and GAPDH (Gapdh) used as reference genes in reverse transcription and polymerase chain reactions. PLoS One. 2012;7:e41659. 10.1371/journal.pone.0041659. PMC3425558 22927912

[2] Jia Y, Chen L, Ma Y, Zhang J, Xu N, Liao DJ. To know how a gene works, we need to redefine it first but then, more importantly, to let the cell itself decide how to transcribe and process its RNAs. Int J Biol Sci. 2015;11:1413–23. 10.7150/ijbs.13436 PMC4671999 26681921

[3] Chapman JR, Waldenstrom J. With reference to reference genes: a systematic review of endogenous controls in gene expression studies. PLoS One. 2015;10:e0141853. 10.1371/journal.pone.0141853 PMC4640531 26555275

[4] de Campos RP, Schultz IC, de Andrade MP, Davies S, Gasparin MS, Bertoni APS, et al. Cervical cancer stem-like cells: systematic review and identification of reference genes for gene expression. Cell Biol Int. 2018;42:139–52. 10.1002/cbin.10878 28949053

[5] Wyckelsma VL, McKenna MJ, Levinger I, Petersen AC, Lamboley CR, Murphy RM. Cell specific differences in the protein abundances of GAPDH and Na(+), K(+)-ATPase in skeletal muscle from aged individuals. Exp Gerontol. 2016;75:8–15. 10.1016/j.exger.2015.12.010 26747222

[6] Garcin ED. GAPDH as a model non-canonical AU-rich RNA binding protein. Semin Cell Dev Biol. 2019;86:162–73. 10.1016/j.semcdb.2018.03.013 29574117

[7] Sirover MA. Pleiotropic effects of moonlighting glyceraldehyde-3-phosphate dehydrogenase (GAPDH) in cancer progression, invasiveness, and metastases. Cancer Metastasis Rev. 2018;37:665–76. 10.1007/s10555-018-9764-7 30209795

[8] Zhu G, Benayed R, Ho C, Mullaney K, Sukhadia P, Rios K, et al. Diagnosis of known sarcoma fusions and novel fusion partners by targeted RNA sequencing with identification of a recurrent ACTB-FOSB fusion in pseudomyogenic hemangioendothelioma. Mod Pathol. 2019;32:609–20. 10.1038/s41379-018-0175-7 PMC6486453 30459475

[9] Agaram NP, Zhang L, Cotzia P, Antonescu CR. Expanding the spectrum of genetic alterations in pseudomyogenic hemangioendothelioma with recurrent novel ACTB-FOSB gene fusions. Am J Surg Pathol. 2018;42:1653–61. 10.1097/PAS.0000000000001147 PMC6608746 30256258

[10] Antonescu CR, Agaram NP, Sung YS, Zhang L, Swanson D, Dickson BC. A distinct malignant epithelioid neoplasm with GLI1 gene rearrangements, frequent S100 protein expression, and metastatic potential: expanding the spectrum of pathologic entities with ACTB/MALAT1/PTCH1-GLI1 fusions. Am J Surg Pathol. 2018;42:553–60. 10.1097/PAS.0000000000001010 PMC5844813 29309307

[11] Dahlen A, Fletcher CD, Mertens F, Fletcher JA, Perez-Atayde AR, Hicks MJ, et al. Activation of the GLI oncogene through fusion with the beta-actin gene (ACTB) in a group of distinctive pericytic neoplasms: pericytoma with t(7;12). Am J Pathol. 2004;164:1645–53. 10.1016/s0002-9440(10)63723-6 PMC1615655 15111311

[12] Kerr DA, Pinto A, Subhawong TK, Wilky BA, Schlumbrecht MP, Antonescu CR, et al. Pericytoma with t(7;12) and ACTB-GLI1 fusion: reevaluation of an unusual entity and its relationship to the spectrum of GLI1 fusion-related neoplasms. Am J Surg Pathol. 2019;43:1682–92. 10.1097/PAS.0000000000001360 PMC6851481 31567194

[13] Nakayama T, Ishida K, Archibald JM. Broad distribution of TPI-GAPDH fusion proteins among eukaryotes: evidence for glycolytic reactions in the mitochondrion? PLoS One. 2012;7:e52340. 10.1371/journal.pone.0052340. PMC3527533 23284996

[14] Takishita K, Patron NJ, Ishida K, Maruyama T, Keeling PJ. A transcriptional fusion of genes encoding glyceraldehyde-3-phosphate dehydrogenase (GAPDH) and enolase in dinoflagellates. J Eukaryot Microbiol. 2005;52:343–8. 10.1111/j.1550-7408.2005.00042x 16014012

[15] Jones CD, Custer AW, Begun DJ. Origin and evolution of a chimeric fusion gene in Drosophila subobscura, D. madeirensis and D. guanche. Genetics. 2005;170:207–19. 10.1534/genetics.104.037283 PMC1449717 15781692

[16] Liaud MF, Lichtle C, Apt K, Martin W, Cerff R. Compartment-specific isoforms of TPI and GAPDH are imported into diatom mitochondria as a fusion protein: evidence in favor of a mitochondrial origin of the eukaryotic glycolytic pathway. Mol Biol Evol. 2000;17:213–23. 10.1093/oxfordjournals.molbev.a026301 10677844

[17] Unkles SE, Logsdon JM, Jr., Robison K, Kinghorn JR, Duncan JM. The tigA gene is a transcriptional fusion of glycolytic genes encoding triose-phosphate isomerase and glyceraldehyde-3-phosphate dehydrogenase in oomycota. J Bacteriol. 1997;179:6816–23. 10.1128/jb.179.21.6816-6823.1997 PMC179613 9352934

[18] Liu X, Wang Y, Yang W, Guan Z, Yu W, Liao DJ. Protein multiplicity can lead to misconduct in western blotting and misinterpretation of immunohistochemical staining results, creating much conflicting data. Prog Histochem Cytochem. 2016;51:51–8. 10.1016/j.proghi.2016.11.001 27908506

[19] Zhang J, Lou X, Shen H, Zellmer L, Sun Y, Liu S, et al. Isoforms of wild type proteins often appear as low molecular weight bands on SDS-PAGE. Biotechnol J. 2014;9:1044–54. 10.1002/biot.201400072 24906056

[20] Yan R, Zhang J, Zellmer L, Chen L, Wu D, Liu S, et al. Probably less than one-tenth of the genes produce only the wild type protein without at least one additional protein isoform in some human cancer cell lines. Oncotarget. 2017;8:82714–27. 10.18632/oncotarget.20015 PMC5669923 29137297

[21] Qu J, Zhang J, Zellmer L, He Y, Liu S, Wang C, et al. About three-fourths of mouse proteins unexpectedly appear at a low position of SDS-PAGE, often as additional isoforms, questioning whether all protein isoforms have been eliminated in gene-knockout cells or organisms. Protein Sci. 2020:29(4):978–90. 10.1002/pro.3823. PMC7096720 31930537

[22] Sun Y, Lou X, Yang M, Yuan C, Ma L, Xie BK, et al. Cyclin-dependent kinase 4 may be expressed as multiple proteins and have functions that are independent of binding to CCND and RB and occur at the S and G 2/M phases of the cell cycle. Cell Cycle. 2013;12:3512–25. 10.4161/cc.26510 PMC3906337 24091631

[23] Liao DZ, Pantazis CG, Hou X, Li SA. Promotion of estrogen-induced mammary gland carcinogenesis by androgen in the male Noble rat: probable mediation by steroid receptors. Carcinogenesis. 1998;19:2173–80. 10.1093/carcin/19.12.2173 9886575

[24] Bollig-Fischer A, Thakur A, Sun Y, Wu J-S, Liao DJ. The predominant proteins that react to the MC-20 estrogen receptor alpha antibody differ in molecular weight between the mammary gland and uterus in the mouse and rat. Int J Biomed Sci. 2012;8:51–63. PMC3614855 23675257

[25] Sun Y, Cao S, Yang M, Wu S, Wang Z, Lin X, et al. Basic anatomy and tumor biology of the RPS6KA6 gene that encodes the p90 ribosomal S6 kinase-4. Oncogene. 2013;32:1794–810. 10.1038/onc.2012.200 PMC3427418 22614021

[26] Yang M, Sun Y, Ma L, Wang C, Wu JM, Bi A, et al. Complex alternative splicing of the smarca2 gene suggests the importance of smarca2-B variants. J Cancer. 2011;2:386–400. 10.7150/jca.2.386 PMC3148773 21811517

[27] Liao DJ, Dickson RB. c-Myc in breast cancer. Endocr Relat Cancer. 2000;7:143–64. 10.1677/erc.0.0070143 11021963

[28] Weingarten-Gabbay S, Khan D, Liberman N, Yoffe Y, Bialik S, Das S, et al. The translation initiation factor DAP5 promotes IRES-driven translation of p53 mRNA. Oncogene. 2014;33:611–8. 10.1038/onc.2012.626 23318444

[29] Xu HJ, Xu K, Zhou Y, Li J, Benedict WF, Hu SX. Enhanced tumor cell growth suppression by an N-terminal truncated retinoblastoma protein. Proc Natl Acad Sci USA. 1994;91:9837–41. 10.1073/pnas.91.21.9837 PMC44912 7937901

[30] Janke C. The tubulin code: molecular components, readout mechanisms, and functions. J Cell Biol. 2014;206:461–72. 10.1083/jcb.201406055 PMC4137062 25135932

[31] Magiera MM, Janke C. Post-translational modifications of tubulin. Curr Biol. 2014;24:R351–4. 10.1016/j.cub.2014.03.032 24801181

[32] Magiera MM, Singh P, Gadadhar S, Janke C. Tubulin posttranslational modifications and emerging links to human disease. Cell. 2018;173:1323–7. 10.1016/j.cell.2018.05.018 29856952

[33] He Y, Yuan C, Chen L, Liu Y, Zhou H, Xu N, et al. While it is not deliberate, much of today’s biomedical research contains logical and technical flaws, showing a need for corrective action. Int J Med Sci. 2018;15:309–22. 10.7150/ijms.23215 PMC5835702 29511367

[34] Brunet MA, Levesque SA, Hunting DJ, Cohen AA, Roucou X. Recognition of the polycistronic nature of human genes is critical to understanding the genotype-phenotype relationship. Genome Res. 2018;28:609–24. 10.1101/gr.230938.117 PMC5932603 29626081

[35] Sofronova AA, Pozdyshev DV, Barinova KV, Muronetz VI, Semenyuk PI. Glycation of glyceraldehyde-3-phosphate dehydrogenase inhibits the binding with Î ± -synuclein and RNA. Arch Biochem Biophys. 2021;698:108744. 10.1016/j.abb.2020.108744. 33385367

[36] Sirover MA. Moonlighting glyceraldehyde-3-phosphate dehydrogenase: post-translational modification, protein and nucleic acid interactions in normal cells and in human pathology. Crit Rev Biochem Mol Biol. 2020;55:354–71. 10.1080/10409238.2020.1787325 32646244

[37] Rodriguez A, Kashina A. Posttranscriptional and posttranslational regulation of actin. Anat Rec (Hoboken). 2018;301:1991–8. 10.1002/ar.23958 PMC6711483 30312009

[38] Terman JR, Kashina A. Post-translational modification and regulation of actin. Curr Opin Cell Biol. 2013;25:30–8. 10.1016/j.ceb.2012.10.009 PMC3578039 23195437

[39] Li R, Shen Y. An old method facing a new challenge: re-visiting housekeeping proteins as internal reference control for neuroscience research. Life Sci. 2013;92:747–51. 10.1016/j.lfs.2013.02.014 PMC3614345 23454168

[40] Cortelazzo A, De FC, Pecorelli A, Belmonte G, Signorini C, Leoncini S, et al. Beta-actin deficiency with oxidative post-translational modifications in Rett syndrome erythrocytes: insights into an altered cytoskeletal organization. PLoS One. 2014;9:e93181. 10.1371/journal.pone.0093181. PMC3966888 24671107

[41] Vigelso A, Dybboe R, Hansen CN, Dela F, Helge JW, Guadalupe GA. GAPDH and beta-actin protein decreases with aging, making Stain-Free technology a superior loading control in Western blotting of human skeletal muscle. J Appl Physiol. 2015;118:386–94. 10.1152/japplphysiol.00840.2014 25429098

[42] Bauer DE, Haroutunian V, McCullumsmith RE, Meador-Woodruff JH. Expression of four housekeeping proteins in elderly patients with schizophrenia. J Neural Transm (Vienna). 2009;116:487–91. 10.1007/s00702-008-0143-3 PMC3734379 19139805

[43] Tzima E, Trotter PJ, Orchard MA, Walker JH. Annexin V relocates to the platelet cytoskeleton upon activation and binds to a specific isoform of actin. Eur J Biochem. 2000;267:4720–30. 10.1046/j.1432-1327.2000.01525.x 10903505

[44] Yu HR, Kuo HC, Huang HC, Huang LT, Tain YL, Chen CC, et al. Glyceraldehyde-3-phosphate dehydrogenase is a reliable internal control in Western blot analysis of leukocyte subpopulations from children. Anal Biochem. 2011;413:24–9. 10.1016/j.ab.2011.01.037 21284931

[45] Yu S, Hwang HE, Yun N, Goldenring JR, Nam KT. The mRNA and protein levels of tubulin and Î²-actin are greatly reduced in the proximal duodenum of mice relative to the rest of the small intestines. Dig Dis Sci. 2015;60:2670–6. 10.1007/s10620-015-3688-7 25976623

